# Overexpression of RRM2 decreases thrombspondin-1 and increases VEGF production in human cancer cells *in vitro *and *in vivo*: implication of RRM2 in angiogenesis

**DOI:** 10.1186/1476-4598-8-11

**Published:** 2009-02-28

**Authors:** Keqiang Zhang, Shuya Hu, Jun Wu, Linling Chen, Jianming Lu, Xiaochen Wang, Xiyong Liu, Bingsen Zhou, Yun Yen

**Affiliations:** 1 Departments of Clinical & Molecular Pharmacology, City of Hope National Medical Center, Duarte, CA 91010, USA; 2 Department of Molecular Medicine, City of Hope National Medical Center, Duarte, CA 91010, USA

## Abstract

**Background:**

In addition to its essential role in ribonucleotide reduction, ribonucleotide reductase (RNR) small subunit, RRM2, has been known to play a critical role in determining tumor malignancy. Overexpression of RRM2 significantly enhances the invasive and metastatic potential of tumor. Angiogenesis is critical to tumor malignancy; it plays an essential role in tumor growth and metastasis. It is important to investigate whether the angiogenic potential of tumor is affected by RRM2.

**Results:**

We examined the expression of antiangiogenic thrombospondin-1 (TSP-1) and proangiogenic vascular endothelial growth factor (VEGF) in two RRM2-overexpressing KB cells: KB-M2-D and KB-HURs. We found that TSP-1 was significantly decreased in both KB-M2-D and KB-HURs cells compared to the parental KB and mock transfected KB-V. Simultaneously, RRM2-overexpressing KB cells showed increased production of VEGF mRNA and protein. In contrast, attenuating RRM2 expression via siRNA resulted in a significant increased TSP-1 expression in both KB and LNCaP cells; while the expression of VEGF by the two cells was significantly decreased under both normoxia and hypoxia. In comparison with KB-V, overexpression of RRM2 had no significant effect on proliferation in vitro, but it dramatically accelerated in vivo subcutaneous growth of KB-M2-D. KB-M2-D possessed more angiogenic potential than KB-V, as shown in vitro by its increased chemotaxis for endothelial cells and in vivo by the generation of more vascularized tumor xenografts.

**Conclusion:**

These findings suggest a positive role of RRM2 in tumor angiogenesis and growth through regulation of the expression of TSP-1 and VEGF.

## Background

Ribonucleotide reductase (RNR) plays an essential role in catalyzing conversion of ribonucleoside diphosphates to the corresponding 2'-deoxyribonucleoside diphosphates, a rate-limiting step in the production of 2'-deoxyribonucleoside 5'-diphosphates (dNTP) required for DNA synthesis and repair [1]. Human ribonucleotide reductase consists of two subunits: RRM1 and RRM2, both proteins are required for enzymatic activity. The cellular RRM1 protein level remains relatively stable throughout the entire cell cycle, whereas RRM2 is only expressed during the late G_1_/early S phase, and degraded in late S phase. The activity of RNR, and therefore DNA synthesis and cell proliferation, is controlled during the cell cycle by the synthesis and degradation of RRM2 subunit [2]. RRM2 itself is a dimmer of two 44 kDa moieties, each containing non-heme iron that stabilizes a unique tyrosyl-free radical required for enzyme activity [3]. Recently cloned p53R2, a new RNR family member, encodes a protein with striking similarity to RRM2, which is induced by UV light, γ-irradiation or adriamycin in a wild type p53-dependent manner [4]. p53R2 directly participates in p53-directed repair of damaged DNA through generating a RNR holoenzyme with RRM1 to synthesize the dNTPs for DNA repair [5].

Many studies have demonstrated that increased RNR enzymatic activity is tightly associated with tumor progression and malignancy. Alteration in the activity of RNR has been identified in a variety of tumor cells obtained from mouse and human tissues [6-8]. Apart from the well-known role in ribonucleotide reduction, several studies have indicated that RRM1 and RRM2 may play opposite roles in controlling malignant progression of tumor cells. Studies had identified that overexpression of both mice R1 and human RRM1 significantly inhibited *in vivo *tumor growth and decreased metastatic potential of tumor through activating PTEN pathway [9-11]. Conversely, overexpression of mouse R2 markedly enhanced cellular transforming potential of various oncogenes; and increased malignant potential of transformed cells [11-13]. We and others had reported that overexpression of human RRM2 could enhance the invasive and metastatic potential of various human cancer cells [14-16]. Our recent study had also shown that the RRM2 protein level in human colon cancer was positively correlated to the metastasis of colon cancer, and elevated RRM2 may serve as a potential biomarker for metastasis of colon cancer [8,14].

Angiogenesis, the formation of new blood vessel from pre-existing vessel, plays a critical role in many physiological and pathological conditions including embryonic development, wound healing, tumor growth and metastasis [17,18]. Angiogenesis is tightly controlled by a balance of pro-angiogenic and anti-angiogenic factors [19]. Among many angiogenesis factors, vascular endothelial growth factor (VEGF) is one of the most critical and specific factors that stimulate both physiological and pathological angiogenesis; the expression of VEGF are induced by growth factors and hypoxia [20]. Whereas, among many antiangiogenic factors, thrombospondin-1 (TSP-1) is a very potent angiogenesis inhibitor, and down-regulation of TSP-1 has been suggested to alter tumor growth by modulating angiogenesis in a variety of tumor types [21]. Oncogenes activation and loss of tumor suppressor genes activity can directly regulate the expression of proangiogenic and antiangiogenic factors in an opposite way thus determining the overall angiogenic activity of tumors [22]. In tumor cells, VEGF is also stimulated by mutated *ras*, v-*src*, c-*fos *and c-*jun etc *[22]. The expression of TSP-1 is upregulated by the tumor suppressor gene, p53, and down-regulated by oncogenes such as myc and ras [20-22].

Tumor angiogenesis is a hallmark of malignancy; it plays an essential role in tumor growth and metastasis [23]. However, the implication of the RRM2 in tumor angiogenesis is not closely studied. Therefore, in the present study, we examined the impact of the RRM2 on tumor angiogenesis through its regulating the expression of proangiogenic factor VEGF, and antiangiogenic factor TSP-1. For the first time, we found overexpression of RRM2 alone reduces TSP-1 expression; induced VEGF expression in tumor cells, resulted in a tumor xenograft with more angiogenic potential and accelerated growth *in vivo*. Our results indicate a positive role of RRM2 in tumor angiogenesis and progression.

## Methods

### Cell Culture

Human oropharyngeal carcinoma KB cells (American Type Culture Collection, Manassas, VA), were cultured in a 5% CO_2 _atmosphere at 37°C on plastic tissue culture plates in DMEM supplemented with 10% fetal bovine serum and 1% penicillin/streptomycin. RRM2-overexpressing KB-M2-D and the mock-transfected KB-V were cultured in the same medium of KB cells with 300 μg/ml G-418 [15]. KB-HURs, a hydroxyurea-resistant clone with an elevated RRM2 protein induced by hydroxyurea, was incubated and maintained in the presence of 1 mM hydroxyurea [15] as another RRM2-overexpressing cells clone in this study. The human prostate adenocarcinoma LNCaP was cultured in RPMI1640 medium containing 10% heat-inactivated fetal bovine serum (FBS; Gibco BRL, Grant Island, NY, USA) and 1 mM sodium pyruvate. For cell culture under hypoxia, cells were grown in a chamber containing 1% O_2_, 5% CO_2 _and 94% N_2 _at 37°C.

### Quantitative reverse transcriptional PCR: q-RT-PCR

Total RNA was extracted from cells by using of Qiagen RNeasy Min Kit. Residual genomic DNA was removed by incubating the RNA with DNase (Qiagen, Valencia, CA). cDNA was synthesized from 1.0 μg of total RNA using the Superscript III first-strand cDNA synthesis kit (Invitrogen, Carlsbad, CA) in a final volume of 20 μl with 0.25 μg random hexamer and 200 Units of Superscript RNase H-reverse transcriptase. The reaction mixture was firstly incubated at 25°C for 5 min, and then followed by incubation at 50°C for 50 min. Quantitative real time PCR was carried out in the ABI Prism 7900 HT Sequence Detection System (Applied Biosystems, Foster City, CA). The reaction mixture of 20 μl consisted of 1× ABI SYBR Green PCR Master Mix, 0.25 μl cDNA and 0.2 μM of each primer. The PCR protocol was: 95°C for 10 min, followed by 40 cycles of 95°C for 15 s, 60°C 1 min. The following primers were used: RRM2, 5'-GCGATTTAGCCAAGAAGTTCAGAT-3' (forward) and 5'-CCCAGTCTGCCTTCTTCTTGA-3' (reverse); TSP-1, 5'-ACGAGGAATGGACTGTTGATAGC-3' (forward) and 5'-ATCAGGAACTGTGGCATTGGAG-3' (reverse). VEGF, 5'-CCAGCACATAGGAGAGATGAGCTT-3' (forward) 5'-TCTTTCTTTGGTCTGCATTCACAT-3' (reverse). Beta-Actin, 5'-ATCTGGCACCACACCTTCTACAA-3' (forward) 5'-GTACATGGCTGGGGTGTTGAAG-3' (reverse).

Relative gene-expression quantification method was used to calculate the fold change of mRNA expression according to the comparative C_t _method using β-actin as an endogenous control. Final results were determined as follows: 2^-(ΔCt sample-ΔCt control)^, where ΔC_t _values of the control and sample were determined by subtracting the C_t _value of the target gene from the value of the housekeeping gene: β-actin. Data was represented as ratio or folds change to control sample.

### RRM2 siRNA Knockdown assay

The human RRM2 gene specific siRNA (sc-36338) and scramble siRNA were purchased from Santa Cruz Biotechnology, Inc. (Santa Cruz, CA). 5 × 10^5^/well KB cells were cultured in six-well plates with 2-mL antibiotic-free growth medium at 37°C in a CO_2 _incubator for 24 hours. siRNA duplex-Lipofectamine was prepared by 6.0 μL of 10 μmol/L RRM2 siRNA or scramble siRNA and 3.0 μl Lipofectamine RNAiMAX (Invitrogen, Carlsbad, CA) in 500 μl serum-free OptiMEM medium (Invitrogen, Carlsbad, CA) according to the manufacturer's instructions, and directly added into the media. 24 or 48 h after transfection, cells was collected [8]. The knockdown of RRM2 was measured by quantitative reverse transcription-PCR (q-RT-PCR) and Western blot.

### Preparation of conditioned medium and ELISA assay

Cells were seeded in 35 mm dishes in 2 ml of growth medium to reach approximately 80% confluence. Cells were washed with PBS 3 times and then were either cultured for 24 h under normoxic or hypoxic condition in 2 ml of serum free medium at 37°C. Cell supernatants were collected, clarified by centrifugation at 2000 rpm for 5 min, and stored at -80°C. Concomitantly, the cell pellets were used in parallel for RT-PCR and total protein concentration. Condition medium for samples of RRM2 knockdown was collected as following. Firstly, cells were cultured in complete medium for 24 h post-transfection under normoxia. Then, cells were further cultured in serum free medium for 24 h under either normoxia or hypoxia (1% O_2_), and the medium was collected. The amount of VEGF in condition medium was measured with a QuantikineTM immunoassay kit (VEGF-ELISA R&D system, Minneapolis, MN, USA) following the manufacturer's instructions. VEGF was expressed as ng of VEGF protein/ml of medium and per mg of total protein.

### Western blot analysis

The mouse monoclonal antibodies against Beta-Actin, RRM2, were obtained from Santa Cruz Biotechnology (Santa Cruz, CA). Mouse monoclonal antibody against human TSP-1 was purchased from Thermo Scientific (Fremont, CA). Western blot was conducted as previously described [8]. Briefly, about 20-μg total protein was separated by 10% SDS-PAGE, transferred to a Nitrocellulose membrane (Bio-Rad Laboratories, CA) and incubated in blocking buffer (1% I-Block reagent and 0.1% Tween 20) with the primary antibody (1:1000 dilution) overnight at 4°C. After 3–5 washes, the membrane was incubated with fluorescent-labeled secondary antibodies (1:8,000 dilutions) for 60 minutes. After sequential washes, membranes were scanned on an Odyssey Infrared Imaging System (LI-COR Biosciences). Signals were densitometrically assessed and normalized to the β-actin signals.

### Human umbilical vein endothelial cell migration assay

Modified Boyden chambers (Millicell PCF, 8 μm pore size; Millipore) were placed in a 24-well plate and coated with 10 μg/ml collagen I (Roche Diagnostics) overnight. After washing with PBS, 0.6 ml of concentrated condition media was placed in the bottom chamber. HUVEC cells were cultured in complete EBM-2 medium (Cambrex Bioscience, MD, USA), and passage 5–6 of HUVEC was used for the experiment. After starvation for 16 h, 2 × 10^5 ^human umbilical vein endothelial cells (HUVEC) were resuspended in 0.3 ml DMEM/0.1% BSA, plated in the top of the transwell chambers, and incubated for 24 h at 37°C with 5% CO_2_. Cells were removed from the upper membrane surface with a cotton tip applicator, washed with PBS, and cells on the lower membrane surface were fixed and stained (Hema 3 Stain System; Fisher Diagnostics). HUVEC migration was quantified by manually counting the number of cells on the inserts under high-power at ×100 magnification. The migration of HUVEC added to the upper compartment of the chamber, was expressed as the number of cells migrated in 10 high-power fields. Values were expressed as the mean ± SD of two independent tests; each test included three individual wells [24].

### *In vitro *proliferation and *in vivo *growth

0.5 × 10^4 ^of KB-V and KB-M2-D cells were replated into wells of 16-well devices compatible with a W200 real-time cell electronic sensing (RT-CES) analyzer and 16× station (Acea Biosciences, San Diego, CA). Cell growth was monitored periodically (typically, every 0.5 or 1 h) for indicated durations via calculation of a "cell index" (normalized impedance) for each well. Unless otherwise indicated, cells from each well of the original six-well plates were replated into four replicate wells for cell index measurement.

The animal protocol for tumorigenicity assay *in vivo *was approved by the Institutional Animal Care and Use Committee of City of Hope Medical Center with RACC protocol number 0750. 6 to 8t-week-old Nod-Scid mice (City of Hope) were subcutaneously inoculated in the right flank with either 5 × 10^6 ^KB-V or KB-M2-D cells resuspended in 100 μL serum-free RPMI. Tumor xenografts appeared approximately 3–6 days following cell injection. Tumor xenograft diameters were measured with digital calipers twice a week, and the tumor volume in mm^3 ^was calculated by the formula: Volume = (width)^2 ^× length/2; and results were presented as mean tumor volume ± SD of two independent experiments. Mouse was sacrificed on 30th day after inoculation. Tumor xenografts were dissected and the final volume and wet weight were determined. Subsequently, they were cut through the median, one part was fixed in formalin and embedded in paraffin, the other part was embedded in Tissue-Tek and snap frozen in liquid nitrogen.

### Immonostainig IHC Microvessel analysis

For immunohistochemical analysis of CD31 (a specific marker of endothelial cells), formalin fixed and paraffin embedded tumor sections were permeabilized with 36 μg/ml proteinase K and stained with a rat anti-mouse CD31 antibody (clone MEC13.3, BD Biosciences), final dilution 1:250. Tyramide signal amplication kits (PerkinElmer Life and Analytical sciences, Inc.) were used with Cyanine 3 red fluorescence. Microvessel density was qualitatively assessed by examining the entire vital cellular zone of the tumors according to the described procedure [25]. Microvessel count was carried out on three fields (×100) chosen within the whole vascularized areas. Any endothelial cell or cluster of endothelial cells positive for CD31 was counted.

## Statistics

Data were collected using an MS-Excel spreadsheet. Data were analyzed using the JMP Statistical Discovery Software version 6.0 (SAS Institute, Cary, NC). Group comparisons for continuous data were done with student's t-test for independent means or two-way ANOVA. Statistical significance was set at *P *< 0.05.

## Results

### Overexpression of RRM2 decreased TSP-1 expression in KB Cells

**Figure 1:**
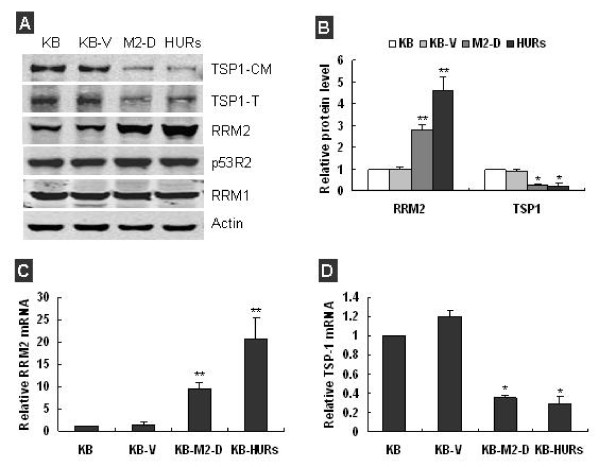
**Overexpression of RRM2 decreased TSP-1 expression in KB-M2-D and KB-HURs cells**. **(A) **Western blot analysis confirmed that only RRM2 was overexpressed in KB-M2-D and KB-HURs; the expression of RRM1 and p53R2 was not significantly modulated in both KB-M2-D and KB-HURs; and the expression of TSP-1 was significantly decreased in KB-M2-D and KB-HURs cells by 60%~70% (TSP1-CM: TSP-1 in concentrated condition medium, TSP1-T: TSP-1 in total protein). **(B) **Representative quantitative densitometric analysis of RRM2 and TSP-1 expression. Signal intensities were normalized to that of β-actin serving as loading control; data was presented as fold of KB. **(C) **q-RT-PCR analysis confirmed that RRM2 mRNA was dramatically overexpressed in KB-M2D and KB-HURs compared with parental KB (**p < 0.01). Values of q-RT-PCR were the mean ± SD of three independent experiments and presented as ratio to the level of parental KB cells. **(D) **q-RT-PCR analysis showed TSP-1 mRNA was significantly decreased in KB-M2D and KB-HURs compared with parental KB (**p < 0.01).

Two human oropharyngeal carcinoma KB cell lines: KB-M2-D, which was forced to overexpress RRM2 protein by gene transfer, and KB-HURs, which was induced to overexpress RRM2 protein by hydroxyurea, were established as reported previously [15]. Overexpression of RRM2 in these two cells was confirmed at both protein (Figure 1a, b) and mRNA level (Figure 1c, *P *< 0.01). The levels of RRM1 (the large subunit of RNR) and p53R2 (the small subunit homologue of RRM2) were not significantly changed in KB-M2-D and KB-HURs as indicated by Western blot analysis (Figure 1a). To answer whether overexpression of RRM2 had a positive effect on tumor angiogenesis, we firstly determined the effect of overexpression of RRM2 on the production of well known pro- and anti-angiogenic factors.

Among many anti-angiogenic factors, TSP-1 is a very critical angiogenic inhibitor; it can directly inhibit endothelial cell migration and proliferation [21,24]. We examined the protein levels of TSP-1 in total protein extracts (abbreviated as TSP1-T in Figure 1a) and equally concentrated condition mediums (abbreviated as TSP1-CM in Figure 1a) from KB, KB-V, KB-M2-D, and KB-HURs. Western-blot analysis displayed that both RRM2-overexpressing KB-M2-D, and KB-HURs cells showed a dramatic decrease in TSP-1 protein compared to control KB and KB-V cells (Figure 1a, b). To investigate whether the decreased TSP-1 protein was due to reduced synthesis at mRNA level, we also measured by q-RT-PCR the TSP-1 mRNA levels in cells. We found, in both RRM2-overexpressing cells, the TSP-1 mRNA levels were significantly decreased (Figure 1d, P < 0.01).

### Overexpression of RRM2 increased VEGF expression in KB Cells

**Figure 2:**
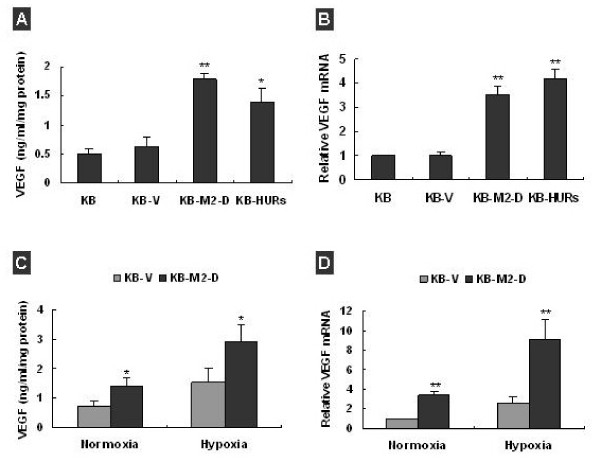
**Overexpression of RRM2 increased VEGF expression in KB-M2-D and KB-HURs cells**. **(A) **VEGF in condition mediums of KB-M2-D and Kb-HURs were increased by 2.4-fold and 2.1-fold respectively. Levels of VEGF in the condition medium were measured with ELISA. Data, presented as ng of VEGF protein/ml of medium and per mg of total protein, was the mean ± SD of 2–3 preparation. **(B) **q-RT-PCR analysis showed that VEGF mRNA was increased in KB-M2D and KB-HURs by 3~4 fold compared with parental KB and KB-V cells. **(C) **Compared with KB-V, KB-M2-D secreted more VEGF under both normoxia and hypoxia. **(D) **q-RT-PCR analysis showed KB-M2D expressed more VEGF mRNA than KB-V under both normoxia and hypoxia, *p < 0.05, **p < 0.01 compared with KB-V.

VEGF is a key mediator of tumor-associated angiogenesis and is thought to support neovascularization by inducing endothelial cell migration and proliferation leading to vascular permeability [18-20]. To answer whether overexpression of RRM2 also effects on VEGF expression in cancer cells, we examined the levels of VEGF in the condition medium of KB cells. By ELISA assay, we found that RRM2-overexpressing KB-M2-D and KB-HURs released about 2.8-fold and 2.2-fold more VEGF than control cells (Figure 2a, *P *< 0.05). The induction of VEGF expression could be regulated at the level of either mRNA or protein. Measured by Q-RT-PCR, we found that in the two RRM2-overexpressing cells VEGF mRNA was significantly increased (Figure. 2b, *P *< 0.01). Hypoxia condition in tumor has been shown to be an important stimulus for VEGF gene expression as well as tumor angiogenesis. To determine the effect of RRM2 on VEGF expression under hypoxia, we compared VEGF mRNA and protein in KB-V and KB-M2-D cells. The results showed that hypoxia (1% oxygen for 16h) upregulated VEGF by ~2 fold at protein levels (Figure. 2c, *P *< 0.05) or ~4-fold at mRNA (Figure. 2d, *P *< 0.01) in both cells. Compared with that of KB-V, the production of VEGF in KB-M2-D was still significantly higher under both normoxia and hypoxia (Figure. 2c, d, *P *< 0.05).

### Knockdown of RRM2 increased TSP-1 and decreased VEGF expression in both KB and LNCaP Cells

**Figure 3:**
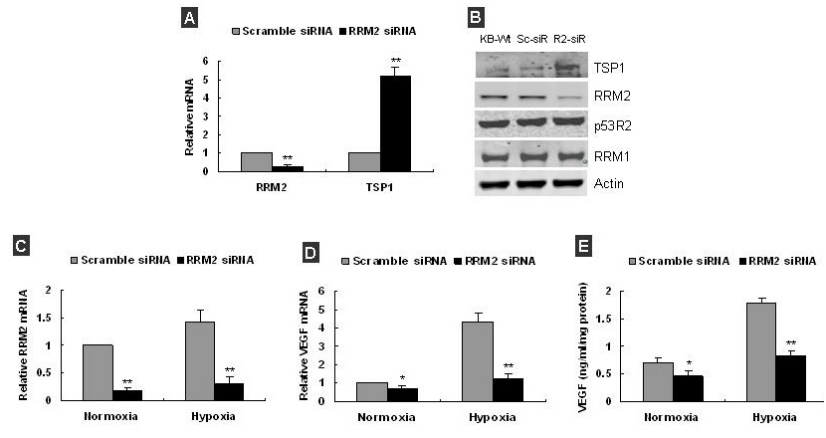
**Knockdown of RRM2 increased TSP-1 and decreased VEGF expression in KB cells**. **(A) **q-RT-PCR analysis showed that RRM2 mRNA was decreased by ~80%, and TSP-1 mRNA was increased by ~5 folds in KB cells at 48 h post-transfection with RRM2 siRNA. **(B) **Western blot analysis showed that TSP-1 protein was significantly increased by ~2.6-fold (KB-W: KB cell, Sc-siR: scramble siRNA transfected KB, R2-siR: RRM2 siRNA transfected KB). **(C) **q-RT-PCR analysis confirmed RRM2 mRNA was significantly decreased in KB cells at 48 h post-transfection under both normoxia and hypoxia. **(D) **q-RT-PCR analysis showed VEGF mRNA in KB cells was decreased after RRM2 knockdown under both normoxia and hypoxia. **(E) **VEGF in condition medium of KB was significantly decreased after RRM2 knockdown under both normoxia and hypoxia. Data was presented as the mean ± SD of 2–3 independent experiments, *p < 0.05, **p < 0.01, compared with scramble siRNA transfected.

Overexpression of RRM2 significantly decreased TSP-1, simultaneously increased VEGF expression in KB cells. To further confirm the implication of RRM2 in the regulation of TSP-1 and VEGF expression, we measured TSP-1 and VEGF expression level, after RRM2 in cancer cells was knocked down by its specific siRNA. Q-RT-PCR and Western blot analysis showed that RRM2 mRNA and protein in KB cells was significantly decreased at 48 h post-transfection of siRNA (Figure 3a, b). RRM2 level at 24 h and 72 h post-transfection of siRNA was also significantly decreased (data not shown). Simultaneously, we measured TSP-1 expression in KB cells, we found TSP-1 mRNA and protein was significantly upregulated at all three points (Figure 3a, b, *P *< 0.05). By using of q-RT-PCR analysis, we confirmed under both normoxia and hypoxia RRM2 mRNA in KB cells was significantly decreased by siRNA transfection (Figure 3c, *P *< 0.01). And knockdown of RRM2 in KB cells moderately decreased VEGF mRNA and protein expression under normoxia, significantly decreased VEGF expression under hypoxia (Figure 3d and 3e, *P *< 0.05).

**Figure 4:**
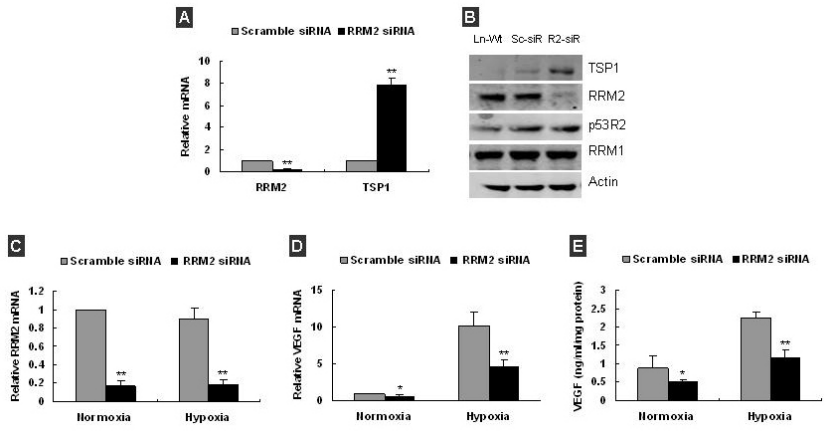
**Knockdown of RRM2 increased TSP-1 and decreased VEGF expression in LNCaP Cells**. **(A) **q-RT-PCR analysis confirmed that RRM2 mRNA decreased by ~80%, and TSP-1 mRNA increased by ~8 folds in LNCaP cells at 48 h post-transfection with RRM2 siRNA. **(B) **Western blot analysis showed that TSP-1 protein was significantly increased by ~3.4-fold (Ln-Wt: LNCaP cell, Sc-siR: scramble siRNA transfected LNCaP, R2-siR: RRM2 siRNA transfected LNCaP). **(C) **q-RT-PCR analysis confirmed that RRM2 mRNA was significantly decreased in LNCaP at 48 h post-transfection under both normoxia and hypoxia. **(D) **q-RT-PCR analysis showed VEGF mRNA in LNCaP cells was decreased after RRM2 knockdown under both normoxia and hypoxia. **(E) **VEGF in condition medium of LNCaP was significantly decreased after RRM2 knockdown under both normoxia and hypoxia. Data was presented as the mean ± SD of 2–3 independent experiments, *p < 0.05, **p < 0.01, compared with scramble siRNA transefected.

It is important to address whether the regulation of RRM2 on TSP-1 and VEGF expression is a cell specific response or not. To answer this question, we then measured the effect of the knockdown of RRM2 on VEGF and TSP-1 expression in human prostate cancer cell: LNCaP, which also contains a wild-type p53. Similar to the change in KB cells, knockdown of RRM2 also significantly increased TSP-1 mRNA and protein (Figure 4a, b, *P *< 0.05). Knockdown of RRM2 in LNCaP cells (Figure 4c, *P *< 0.01) significantly decreased VEGF mRNA and protein under both normoxia and hypoxia conditions (Figure 4d, e, *P *< 0.05). The decreased VEGF in LNCaP was more pronounced under hypoxia condition. The data indicates that the regulation of RRM2 on TSP-1 and VEGF may not be a cell type specific but a common event; and a common mechanism may responsible for this regulation.

### RRM2-overexpressing KB-M2-D cells showed accelerative tumor growth *in vivo*

**Figure 5:**
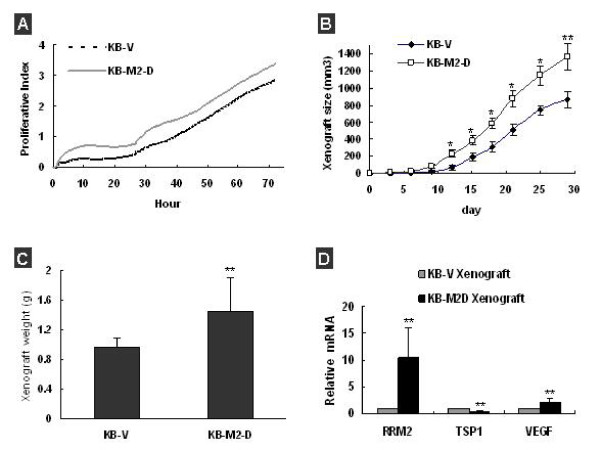
**Overexpression of RRM2 promoted the growth of KB-M2-D *in vivo***. **(A) **Typical RT-CES traces described that KB and KB-M2-D proliferated in an equal rate. CI was recorded every half hour. Each trace for each cell was an average of 4 replicates. **(B) **Tumor growth curves showed that RRM2 significantly promoted KB-M2-D *in vivo *growth. **(C) **Overexpression of RRM2 increased tumor xenografts size of KB-M2-D at the end of experiment. **(D) **q-RT-PCR analysis confirmed that RRM2, VEGF and TSP-1 mRNA was significantly different in KB-V and KB-M2 xenografts. *p < 0.05; **p < 0.01, compared with Kb-V. Data expressed was the mean ± SD of 12 mice of two independent experiments.

In order to determine whether overexpression of RRM2 had an effect on cell *in vitro *proliferation in KB cells, RT-CES cell proliferation assays (Acea Biosciences, San Diego, CA) were performed with KB-V and KB-M2D. RT-CES traces displayed that proliferation of KB-M2-D did not demonstrated significant differences with that of control KB-V over a period of 72 h (Figure 5a). Next, we assessed the effect of RRM2 on tumor *in vivo *growth. The KB-V and KB-M2-D were inoculated subcutaneously in the hind flank of non-SCID mice. The tumor xenografts were measured on a regular basis for 30 days. We found ~5–6 days after inoculation the growth of KB-M2-D xenograft begun to display a significant larger mass than that of xenografts KB-V (Figure 5b, *P *< 0.05). Correspondingly, compared with that of KB-V, the average weight of KB-M2-D xenografts was also significantly increased by ~50% (Figure 5c, *P *< 0.01). q-RT-PCR analysis confirmed that the expression of RRM2 and VEGF was significantly higher in KB-M2-D xenografts than these in KB-V xenografts, TSP-1 in KB-M2-D xenografts was significantly lower (Figure 5d, *P *< 0.01). Those data confirmed that overexpression of RRM2 promotes cancer cells *in vivo *growth.

### Overexpression of RRM2 promoted angiogenic potential of tumors *in vitro *and *in vivo*

Tumor angiogenesis is controlled by a balance of pro-angiogenic and anti-angiogenic factors [18-20]. We questioned whether the increased production of angiogenic factors VEGF and the parallel reduction of the inhibitor TSP-1 by RRM2 would affect the angiogenic potential of tumors. In vitro, we measured the ability of condition medium collected from KB-V and KB-M2-D cell lines to affect endothelial cell motility, a crucial event of the angiogenic process, known to be modulated by the VEGF and TSP-1 etc [18-20]. Using the Boyden chamber assay, we identified the HUVEC attracted by condition medium of KB-M2-D was 78.3 ± 10.7 (mean ± SD), and that attracted by condition medium of KB-V was 25.6 ± 6.4; statistic analysis indicated that the condition medium of the KB-M2-D were significantly more active in stimulating the migration of HUVEC cells in comparison with that of control KB-V cells (*p < 0.01*).

**Figure 6:**
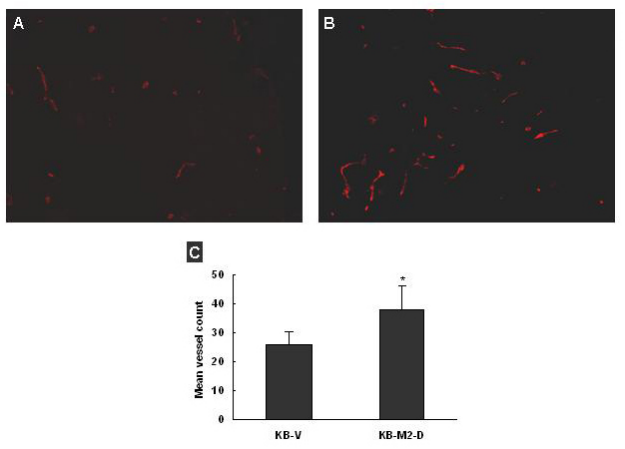
**CD31 immunohistochemistry assay displayed that overexpression of RRM2 resulted in more vascularized tumor xenografts**. Representative immunohistochemical assay of vascularization of tumor xenografts generated in vivo by **(A) **KB-V and **(B) **KB-M2-D cells (×100). **(C) **Semi-quantitative analysis showed that CD31 staining was significantly increased in KB-M2D tumor xenografts compared with that of KB-V, *p < 0.05. Values expressed were the mean ± SD of 8 mice of two independent experiments.

To evaluate the effect of RRM2 on the *in vivo *angiogenic potential of tumor cells, we measured the microvascular density in KB-V and KB-M2-D xenografts. The tumor sections were used for immunohistochemical analysis for the endothelial marker: CD31 and the number of CD31 positive vessels per tumor was assessed. The tumor xenografts derived from KB-M2-D had a significantly higher number of CD31 positive vessels as compared to the vector control KB-V cells (Figure 6a, b). Mean vessel counting per field was 26 ± 4.2 for KB-V (n = 8) and 37.8 ± 8.4 for KB-M2-D (n = 8), the difference were significant in statistic (Figure 6c, *P *< 0.01). Our data indicated that overexpression of RRM2 positively affects angiogenesis *in vivo*.

## Discussion

RRM2 is well-known as small subunit of RNR, a rate-limiting enzyme for dNDP synthesis required for DNA replication [1-3]. High RNR enzymatic activity is associated with tumor progression and resistance to various cellular stressors such as chemotherapeutic agents and ionizing radiation [6-8]. Increased RRM2 and RNR enzymatic activity were reported to present in highly metastatic tumor cells and tissues [9-11]. Therefore, RRM2 is also an important therapeutic target for DNA replication-dependent diseases such as cancer. Recently, many studies have demonstrated that RRM2 plays additional roles in determining the malignant potential of tumor cells. For example, elevated expression of RRM2 has been found to increase the drug-resistant properties of cancer cells and significantly enhance the invasive potential of many human cancer cells [3,9,10,15], whereas knockdown of RRM2 expression results in the reversal of drug-resistance and suppressed tumor growth, and decreased metastasis potential [14,16,25]. Studies have identified that overexpression of both RRM1 and RRM2 leads increased RR activities and expended dNTP pools [[9,13,15], and [26]]. Interestingly, two recent studies have the studies found that overexpression of RRM1 suppresses invasion and metastasis formation of tumors through induction of PTEN pathway [9-11]. These data suggest two subunits of RNR play opposing roles in tumor progression and malignancy; beside they cooperate in dNTP production.

Tumor angiogenesis is necessary for the growth and metastasis of many tumors. Potential of angiogenic switch is a critical character of tumor malignancy [17-19]. Tumor angiogenesis and metastatic formation are intrinsically connected [20,27]. Accumulating evidences support RRM2 possesses oncogene-like properties; it plays a potential role in tumor malignancy and metastasis. In this study, we therefore focused on the effect of RRM2 on tumor angiogenesis. We investigated the effect of overexpression of RRM2 on the production of angiogenesis regulatory factors in human cancer cells. Our study demonstrated that overexpression of RRM2 decreased the production of TSP-1 and increased that of VEGF in KB cells. Because hypoxia is a critical factor regulating VEGF expression through stabilizing its transcriptional factor HIF-1 protein and it is also an essential factor for tumor development [28]. We also examined the effect of RRM2 on VEGF expression under hypoxia condition. The data demonstrated that under hypoxia condition overexpression of RRM2 further increased VEGF protein in KB cells. To further confirm the regulation, we measured TSP-1 and VEGF expression in KB and LNCaP cells after RRM2 knockdown by its specific siRNA. Contrast to overexpression of RRM2, we found knockdown of RRM2 expression caused an increased TSP-1 and decreased VEGF expression in both KB and LNCaP cells. Considering the potential therapeutic application of RNA interference for RRM2 such as siRNA, antisense oligo-nucleotide [16,25], our above finding is potentially important, and it also indicates that the therapy based on RNA interference for RRM2 may potentially inhibit tumor angiogenesis beside its known inhibitory effects [16,25]. The changes in the expression of angiogenic factors: TSP-1 and VEGF induced by RRM2 in tumor cells did result in increased angiogenic activity *in vitro*, as shown by the increased chemotactic activity for endothelial cells. Moreover, tumors generated *in vivo *by RRM2-overexpressing cells showed a higher vascular density compared to controls. We supposed that higher vascular density might partially contribute to growth advantage of RRM2-overexpressing cancer cells. Consistent with what we expected, the increased vascularization caused by overexpression of RRM2 did confer a growth advantage to RRM2-overexpressing cancer cells.

VEGF is significantly associated with poor outcome in human various malignancies [29,30]. The increased production of VEGF is significant for various cancers development. Exposure of tumor cells to hypoxia is a common finding in solid tumors. Hypoxia induces a myriad of adaptive changes within tumor cells, which result in increased anaerobic glycolysis, new blood vessel formation and genetic instability [28]. Consistently, a previous study by Graff et al [31] also found that elevated RNR caused by overexpression of RRM2 could overrule long-lasting arrest of DNA-synthesis after severe hypoxia insult, and cancer cells with elevated RRM2 were more resistant to hypoxia. TSP-1 is an important inhibitor of angiogenesis, and its suppression is crucial for the angiogenic switch in many tumor models [[32,33], and [34]]. Additionally, TSP-1 is an inhibitor of invasion; it inhibits the activity of matrix metalloproteinase-9 (MMP9), which causes release of vascular endothelial growth factor sequestered in the extracellular matrix, thereby increasing invasion potential [35]. Coincidentally, a recent study of Duxbury et al [36] also found that overexpression of RRM2 increased pancreatic adenocarcinoma cellular invasiveness and MMP-9 expression.

The earlier study by Wright et al [12,13] reported that mouse RRM2 synergize with a variety of oncogenes including h-ras, h-rac, v-src, a-raf, c-myc, etc in cellular transformation and tumorigenic potential to normal fibroblast cells through a variety of signal pathways. For example, cooperation with Ras, overexpression of mouse RNR small subunit R2 significantly increases membrane-associated Raf-1 protein and MAPK-2 activity, finally further activates MAPK pathway [13]. Oncogenes not only promote aberrant cellular mitogenesis, but also have an important impact on tumor formation and growth through an indirect mechanism, namely, by driving tumor angiogenesis [20-22]. Oncogenes such as v-*myc*, c-*jun*, v-*src *h-ras etc down-regulate the TSP-1 expression [20-22]. Additionally, some of them – for example, h-ras and c-myc – also significantly up-regulate VEGF expression [34]. Contrast to oncogenes, tumor suppressor genes such as p53, Rb and PTEN etc stimulate TSP-1 expression [20-22]. Interestingly, our previous study found that in cancer cells, even without any stress RRM2 and p53R2 are bound to wild-type p53 protein. In response to UV irradiation, RRM2 and p53R2 dissociate themselves from p53 and form RNR with RRM1 [37]. It is interesting to further examine whether elevated RRM2 has any impact on p53 function through the protein-protein interaction. Coincidently, we found that knockdown of RRM2 leads a significantly elevated p53 protein in both KB and LNCaP (unpublished data); we are focusing on the correlations among these proteins. Since p53 acts as a negative controller of angiogenesis, and the loss of functional p53 results in high VEGF and low TSP-1 production, with consequent increase in angiogenic activity [38]. The mechanisms through which RRM2 regulate TSP-1 and VEGF expression requires further investigation.

## Conclusion

Overexpression of RRM2 in KB cell suppressed TSP-1 expression, and increased VEGF expression. Conversely, attenuating RRM2 expression via siRNA resulted in a significant increased TSP-1 expression and decreased VEGF production in both KB and LNCaP cells. Our study demonstrated that overexpression of RRM2 substantially promoted the *in vivo *growth of tumor cells, and positively affected the overall angiogenic activity of tumor cells. Knockdown of RRM2 by RNA interference such as siRNA may potentially inhibit cancer angiogenesis.

## Competing interests

The authors declare that they have no competing interests.

## Authors' contributions

KZ designed experiments, drafted the manuscript and performed siRNA knockdown, real time-PCR, Western-blot, ELISA tests. SH was responsible for cell culture, cell proliferation and condition medium preparation. JL was responsible for CD31 immunostaining. LC and XW participated in PCR analysis and data collection. JW was responsible for tumor xenograft experiment and participated in discussion. XL and BZ participated in discussion and manuscript preparation. YY conceived the study and revised the manuscript. All authors read and approved the final version of the manuscript.
